# Returning the student to school after concussion: what do clinicians need to know?

**DOI:** 10.2217/cnc.15.4

**Published:** 2015-08-06

**Authors:** Sean C Rose, Kelly A McNally, Geoffrey L Heyer

**Affiliations:** 1 Division of Pediatric Neurology, Nationwide Children’s Hospital, 700 Children’s Drive, Columbus, OH 43205, USA; 2 Department of Neurology, The Ohio State University, Columbus, OH, USA; 3 Division of Pediatric Psychology & Neuropsychology, Nationwide Children’s Hospital, 700 Children’s Drive, Columbus, OH 43205, USA; 4 Department of Pediatrics, The Ohio State University, Columbus, OH, USA

**Keywords:** academic accommodations, adolescent, concussion, mild traumatic brain injury, pediatric, school, TBI

## Abstract

Participation in school is vital to a child’s academic and social development. Following concussion, returning the student to school can pose several challenges for families, healthcare providers and school personnel. The complex constellation of postconcussion symptoms can impair learning and can make the school environment intolerable. Research evidence to guide the return to school process is lacking, but protocols have been proposed that outline a gradual reintroduction to school with academic accommodations tailored to the student’s specific symptoms. Key medical and school personnel must understand their respective roles to optimize the process. This review of the current literature examines the available data and expert recommendations that can support a student’s successful return to school following concussion.

Between 2009 and 2014, youth advocates and lawmakers successfully enacted state legislation across the USA and the District of Columbia that targets various aspects of sports-related concussion in youth, including mandating that youth athletes be removed from play when a concussion is suspected, allowing return to play only after clearance by a healthcare provider, and supporting concussion education for the athletes, parents and coaches [1]. These state laws promote concussion safety and awareness in youth sports, but they do not address the potentially greater challenge related to concussion among school-aged patients: the return to learning.

Participation in school is vital to a child’s academic and social development. Returning the student to school following concussion poses several challenges for families, healthcare providers and school personnel. The complex constellation of cognitive, emotional and somatic postconcussion symptoms can impair learning and interfere with school attendance. The cognitive exertion (or ‘cognitive overexertion’) [2] related to classwork and testing may exacerbate existing concussion symptoms or cause the re-emergence of symptoms that had previously resolved. Difficulties with memory and attention following concussion can affect a child’s ability to learn new things [3], while symptoms such as headache and sensitivities to bright lights and loud noises can make the classroom environment intolerable. Successfully returning the student to school requires that the burden of postconcussion symptoms be balanced against the academic and psychological consequences of prolonged school absence [4]. In most cases, this balance can be achieved when the student and family, the school and the healthcare provider are able to communicate effectively, monitor postconcussion symptoms, implement academic accommodations according to symptoms, adjust these supports when symptoms change and have reasonable expectations about academic progress while symptoms are present. Concussion education is important for all involved. Healthcare providers and school personnel must understand their respective roles in supporting the student during the transition back to learning.

While research evidence to guide the return to school process is sparse, management strategies and protocols have been proposed for healthcare providers and school personnel [4–7]. This review of the current literature examines some of these recommendations and discusses potential school accommodations for the returning student. Much like the protocols for returning a youth athlete to play, each student should be managed individually, according to his or her symptoms, through each phase of the return to school process [4,5]. Safety and efficacy always supersede expediency. Not all students will adjust quickly to the school environment; some will have protracted postconcussion symptoms. We believe that these are the children who will benefit most by careful planning, close monitoring and effective collaborations among the medical and school teams.

## The theory & evidence for cognitive rest

Cognitive rest is recommended as a cornerstone of concussion management [8]. This consensus recommendation includes physical rest also and is presented in the context of athletes returning to play. The notion of cognitive rest originates from what is known about the neurometabolic changes induced by a concussive injury. During the acute postconcussion period, a cellular energy crisis develops from shifts in ion gradients, glutamate release, depletion of ATP and reversible alterations in cerebral blood flow [9–11]. Theoretically, in the setting of an already overwhelmed metabolic landscape, the increase in cerebral metabolic demands related to cognitive activities with school work could worsen postconcussion symptoms and delay recovery.

Evidence from some studies suggests that cognitive rest can improve concussion outcomes. High school and college-aged athletes who were given recommendations for cognitive and physical rest at various times following concussion (1–7 days, 8–30 days and >30 days) all had symptom improvements and better performance on computerized neurocognitive testing after the rest period, but the study did not include control groups for comparison [12]. Brown and colleagues demonstrated the independent influence of cognitive rest on concussion outcomes by dividing 335 patients referred to a sports concussion clinic, aged 8–23 years, into four quartiles based on self-reported levels of cognitive activity [13]. Those with the highest levels of cognitive activity had the longest postconcussion symptom durations. There were no differences between the other three quartiles, suggesting that moderate cognitive rest may be as effective as stricter forms of rest in terms of therapeutic benefit. The self-report and choice of adherence to certain levels of cognitive activity may have biased outcomes in this nonrandomized study. Similarly, a retrospective study found that athletes who engaged in more activity after concussion demonstrated worse performance on neurocognitive testing than those who engaged in moderate activity levels [14]. Extrapolating from these studies, it is possible that returning to school, part time or full time, with appropriate academic accommodations would allow the student to enjoy the reintroduction of premorbid activities without prolonging overall symptom duration. Research evidence is not available, however, to define the most appropriate academic accommodations or to guide how quickly students can return to school or be advanced through levels of cognitive activity.

Some concussion experts question whether rest, or at least prolonged rest, is the best medicine. Silverberg and Iverson point out that other health conditions can be worsened by prolonged rest and inactivity [15]. They recommend gradually resuming premorbid activities as soon as tolerated and avoiding complete rest beyond 3 days in all concussion patients. Depressed mood and fatigue can emerge when healthy individuals are deprived of their usual exercise activities [16]. Cognitive rest did not improve postconcussion recovery among patients aged 8–26 years when cognitive activity levels were assessed independently of physical activity levels [17]. However, symptom duration was not known for approximately 27% of the cohort, and those whose symptoms had not resolved by the end of the study period were excluded, limiting the generalizability of the results. Last, Thomas and colleagues randomized emergency department patients with concussion, aged 11–22 years, based on rest recommendations: 5 days of strict rest (intervention) versus 1–2 days of rest followed by a stepwise increase in activity (standard care) [18]. Both groups reported engaging in decreased (but equal) levels of physical activity, while self-reported levels of cognitive activity differed. As expected, the intervention patients who were advised strict rest had less school attendance than the standard care group. There were no differences in neurocognitive or balance outcomes between groups, but the intervention group reported more daily postconcussion symptoms and slower symptom resolution than the patients who were advised less cognitive rest. While not directly analyzed, the increase in school attendance did not appear to worsen postconcussion symptoms.

Although the risks and benefits of cognitive rest following concussion still remain unclear, until definitive guidance is available, some degree of cognitive rest should be recommended during the acute recovery period. Cognitive rest includes avoiding or reducing reading, computer use, texting, watching television or movies, playing video games and similar mental activities. Complete cognitive rest (i.e., the absence of cognitive activity) is impractical, and attempts at isolating the student to prevent all forms of external stimulation is not advised. Rather, the goal of rest is to modify those activities that trigger or exacerbate postconcussion symptoms. Gioia describes the concept of ‘not too little, not too much’ in guiding recommendations for cognitive restrictions [4]. Students can work up to their symptom threshold, but should not exceed it. As symptoms improve, cognitive activities can be advanced. Cognitive rest recommendations should incorporate school attendance and classwork. Returning to school with appropriate accommodations, even when cognitive symptoms are present, is probably beneficial by allowing the gradual reintroduction of premorbid school activities with minimal risk of cognitive overexertion. When numerous school absences occur, students can suffer from social isolation and mounting academic stress, both of which can have a negative psychosocial effect [4]. The challenge lies in balancing the need for cognitive rest with the timely return to school and resumption of classwork.

## Returning to school

When a school-aged child sustains a concussion, several school-related questions need to be answered: when should the student return to school? Should the curriculum or the workload be altered? Which accommodations (if any) should be implemented? Which school activities (if any) should be restricted? Evidence-based guidelines do not exist to answer these questions, but expert recommendations are available. Before discussing specific accommodations and protocols, it can be helpful to identify key personnel in the process and to define their roles in supporting the student’s return to a learning environment.

### The student

A student with concussion may enter the medical system in a variety of ways, including through an on-field evaluation by a school physician, through the emergency department or urgent care center, through the primary care provider or through referral to a subspecialist. However, to enter the medical system, first a concussion must be suspected. When concussion signs are absent, recognition that an injury occurred relies, to some extent, on student self-report. But not all students disclose all concussions [19–23]. Several factors play a role in concussion reporting. Athletes may perceive pressure from coaches, teammates, parents and fans to continue playing after a head impact. Athletes who experience pressure from multiple sources are less likely to report their head injuries [20]. In a review of 30 published studies, Kerr and colleagues categorized the reasons for concussion nondisclosure: intrapersonal (e.g., lack of knowledge, internal pressure, gender, concussion history); interpersonal (e.g., other’s concussion knowledge and attitudes, external pressure, external support); environment (e.g., access to concussion prevention materials, sports culture); and policy (e.g., concussion-related legislation) [24]. The authors note that past researchers had focused more on the intrapersonal and interpersonal factors, placing less emphasis on the environment and policy categories.

Concussion education is imperative for students, especially student athletes. Studies have found that student athletes generally lack knowledge about concussion symptoms and concussion risks, and this lack of knowledge may lead to premature return to play [19,23,25–27]. Each state has the jurisdiction to mandate a concussion education program or to establish minimum standards of instruction for student athletes and their parents [28]. Enactment of concussion-related state laws may have improved concussion reporting by some student athletes [29,30]. A few nationally recognized educational programs (listed below) have been developed to improve student education and awareness about concussion [28].

### The medical team

When the student enters the medical system, the medical team comprises all clinicians and healthcare professionals who provide initial or ongoing concussion management, including medical recommendations related to school. The types of providers authorized to manage concussions varies from state to state. For example, in many states the athletic trainer is allowed to assess and clear an athlete following concussion. The healthcare professional who provides the initial concussion assessment is charged with determining immediate activity restrictions and whether the student should attend the next school day or not. The emergency department physicians and other first-line providers should give initial guidance about return to school until the student can be evaluated by a primary care clinician or a concussion specialist who will assume the management role. Minimally, the first-line provider should educate the family about concussion risks and document all activity and school recommendations in writing for the family, school and other medical team members. Unfortunately, chart reviews suggest that few emergency department and general healthcare providers are giving written recommendations about cognitive rest following concussion, although when surveyed the majority of general pediatricians acknowledged that rest is an important part of concussion management [31,32]. The clinician who assumes medical care after the initial encounter then monitors symptoms and communicates recommended adjustments in school attendance and academic accommodations to the family and the school team. Some students return to school and activity prematurely and experience an exacerbation of concussion symptoms [33].

Unfortunately, concussion training varies among health professionals, and management strategies may differ [34]. While pediatric primary care and emergency medicine providers acknowledged their roles in concussion management and family education about concussion, they identified several barriers including inadequate training to educate, inadequate time to educate and a lack of infrastructure to systematically diagnose and manage these patients [35]. Treatment recommendations for concussion, including recommendations for resuming school, were not consistently documented by general pediatricians in the electronic medical record [31]. Zuckerbraun and colleagues, however, demonstrated that a simple intervention in an emergency room setting can improve family education and school management following concussion [36]. The researchers compared families pre- and post-implementation of a modified acute concussion evaluation (ACE) tool which included home discharge instructions and a Return to School form. The return to school form alerted school personnel of concussion symptoms and listed potential supports that may be needed. The ACE group had better parental recall of concussion education, activity restrictions and school management recommendations, and a greater number of children in the intervention group received academic accommodations [36].

### The school team

The school team facilitates the student’s return to school following concussion. The team comprises all school personnel who monitor students with concussion, evaluate their symptoms, determine their academic supports and accommodations or communicate about symptoms or supports with families or medical teams. Potential participants include the school principal and vice principal, teachers, school nurses, school psychologists, athletic trainers, coaches, school counselors and social workers [2,6], but the team composition may differ by school. Some schools may designate certain faculty members to act as ‘case managers’ for students, while other schools have teams in place that respond to all student complaints and concerns (including those related to concussion) and implement interventions [6,37].

Schools vary in their strategies for monitoring and accommodating students following concussion. In a survey of high school principals in the state of Ohio, the school personnel designated to monitor students with concussion differed by school [37]. Most principals responded that they were willing to provide students with short-term academic accommodations, but 30.1% required a medical provider’s note prior to making any academic changes. Some of the differences in management were related to school resources and respondent knowledge and training about concussion [37]. Athletic trainers are ideally poised to educate school faculty and to coordinate the gradual return to school for student athletes [7], but access to athletic trainers differs across school districts [37,38]. The school team needs to facilitate school return for all students with concussion, regardless of athlete status. In the survey, athletic trainers were identified most frequently as the school designee who communicates with the medical team when the student with concussion is an athlete, while guidance counselors and school nurses were more frequently identified as designees when students with concussion were not athletes [37]. In a separate survey assessing school nurses’ perceptions and familiarity with academic accommodations relevant to youth concussion, school nurses had more numerous personal experiences with student concussion and more knowledge about academic accommodations for these students when an athletic trainer was employed at their school [39]. Nurses employed at a single school were more familiar with academic accommodations for concussion than nurses who worked at multiple schools. Given proper training, a variety of school personnel could assume the role of monitoring the students with concussion, communicating with their parents and medical providers and facilitating accommodations.

Although state legislation pertaining to concussion in youth sports has been passed across the USA, only those school personnel who are directly involved with student athletics are required to participate in the education effort in most states. However, it is important that members of the school team who are not coaches or referees have adequate concussion education and training to monitor students’ symptoms and to adjust accommodations appropriately. High school principals who had some sort of concussion training within 1 year of survey were more likely to provide or support concussion training for school faculty who were not directly involved in youth sports [37]. Glang and colleagues developed an internet-based intervention (Brain 101: The Concussion Playbook), designed to promote concussion education for members of the school community who are not directly involved in youth sports, to provide guidelines for creating a school team for concussion, and to describe strategies for supporting students in the classroom [40]. Twenty-five participating high schools were randomly assigned to the Brain 101 intervention or to a nonintervention control group. Students at Brain 101 schools who had concussions during the study received more varied academic accommodations than students in control schools. The researchers concluded that the intervention can help schools create a comprehensive school-wide concussion management program. Additionally, the CDC provide several online educational resources that are suitable for teachers and school personnel, including the CDC Heads Up to Schools: Know your Concussion ABCs ‘Returning to School after Concussion: A Fact Sheet for School Professionals’ [41] and ‘Helping Students Recover from a Concussion: Classroom Tips for Teachers’ [42]. The Heads Up: Concussion in Youth Sports program offers educational support for coaches, healthcare professionals, educators, parents and student athletes [43].

### Common themes guiding the return to school process

Gioia reviewed guidelines and recommendations designed to help transition students with other medical conditions (migraines, epilepsy, diabetes and asthma) from illness back into school [4]. Using these materials, he developed six common themes that apply to students with concussion as they re-enter school, summarized below:

- Medical providers and key school personnel should be appropriately trained for their specific role in managing the returning student;
- An initial medical evaluation should be done to assess symptom status and to guide restrictions and accommodations;
- A school team should be formed that is skilled in translating the student’s symptom profile into academic supports/accommodations;
- A student symptom profile and management plan should be communicated directly by the medical provider to the school team;
- Periodic in-school monitoring of symptoms should be done, and the school supports should be adjusted accordingly by the school team;
- Regular, ongoing communication about symptom status and concerns should occur between the student and family, the medical provider and the school team.

Additionally, we suggest that the school team provide the family with a written plan describing the academic accommodations available at their school and the contact information for key school personnel.

## Academic accommodations & supports

A balance must be struck between cognitive rest, postconcussive symptoms and the return to school following concussion. Returning to the school environment and resuming classwork too early may not be tolerated and may even worsen some postconcussion symptoms; yet, excessive activity restriction and postponement of the return process can have a negative effect on recovery caused by anxiety and depression from loss of academic standing, inactivity and social isolation [16]. Once cognitive activities are tolerated for at least 30–45 min at home, we and others encourage attempts at school return, even though the child may not be ready to learn [4,6]. Addressing symptom triggers, attending partial school days and allowing periods of rest while at school can help during this important transition period. The aim is to provide adequate supports and accommodations for the student, based on his or her specific symptom profile, until concussion symptoms improve and learning can resume. Academic accommodations should remain fluid, adjusted according to changes in symptoms.

### The acute concussion

After assessing the acute postconcussion symptoms, the healthcare provider determines cognitive and physical activity restrictions, which include recommendations about when return to school can be attempted. Prior to school return, written recommendations for individualized academic accommodations should be given to the family and the school team. Other publications offer examples of physician letters with potential academic accommodations [4,44]. We include a simplified sample letter that the healthcare provider can complete quickly in the office setting (Supplementary Material). Providing families with recommendations about school accommodations increases the likelihood that they will receive support from their school [36].

Upon school return, or prior to return, the school team establishes a cohesive support and accommodation plan based on the student’s needs and the medical provider’s requests. This can be achieved through a formal meeting with the student and parents, teachers and at least one school administrator. Other school personnel may also be involved including the athletic trainer, school nurse or school counselor. The purpose of the meeting is to determine how to implement any recommended accommodations based on the school’s resources. The student’s symptoms are reviewed in order to establish a reference point for future comparison. The meeting should generate a written school plan with accommodations and the contact information for key school personnel. Given the limited resources of most schools and the brief nature of postconcussion symptoms in most students, a formal meeting may not be feasible (or necessary). Alternatively, a written plan can also be generated and shared among families, school personnel and healthcare providers without a formal meeting.

### Return to school protocols

Several published protocols describe stages of cognitive and academic activities that the student can progress through in order to return to full, unrestricted school participation [4–5,45]. Gioia recommends a staged return to school protocol (Table 1), analogous to the protocols used for return to play [4]. The protocol begins with school absence and cognitive rest and gradually progresses through partial school days with maximal supports, full days with maximal supports, full days with moderate supports, full days with minimal supports and then normal school days. Importantly, not all students will need to advance through each independent stage of the protocol; some will be ready to return to a full academic day quickly after their injury. In contrast to return to play protocols, some flexibility, based on symptom burden, is reasonable. Without data to show its efficacy, the ideal duration of school absence is not known and will depend on the individual student. Gioia recommends 1–2 days [4], whereas DeMatteo and colleagues recommend at least 1 week [5]. We find that for most students, 1–2 days of rest is appropriate and does not result in a significant amount of missed schoolwork. The ability to tolerate cognitive activity at home serves as a reasonable indicator of when the student is ready to attempt school return. Some providers recommend that the child tolerates 30–45 min of cognitive activity at home [4,6], while others advocate for 2 h of total daily activity that can be divided up [45]. Alternatively, the student can be returned to the school environment before cognitive activities are tolerated, especially if several school days have been missed; once in school, the support team determines when he or she is ready to start participating in classwork and learning again [5].

**Table 1.** **Protocol for return to full academic participation.**

**Stage**	**Description**	**Activity level**	**Criteria to move to next stage**
0	No return, at home	Day 1: maintain low-level cognitive and physical activity. No prolonged concentration. Cognitive readiness challenge: as symptoms improve, try reading or math challenge task for 10–30 min; assess for symptom increase	To move to stage 1: Student can sustain concentration for 30 min before significant symptom exacerbation and Symptoms reduce or disappear with cognitive rest breaks.

1	Return to school, partial days (1–3 h)	Attend one to three classes, with interspersed rest breaks as needed. Minimal expectations for productivity. No tests or homework.	To move to stage 2: student symptom status improving, able to tolerate 4–5 h of activity with two to three cognitive rest breaks built into school day

2	Full day, maximal supports required throughout the day	Attend most classes, with two to three rest breaks (20–30 min), no tests. Minimal homework (<60 min). Minimal-to-moderate expectations for productivity	To move to stage 3: number and severity of symptoms improving, needs only one to two cognitive rest breaks built into school day

3	Return to full day, moderate supports provided in response to symptoms during the day	Attend all classes with one to two rest breaks (20–30 min); begin quizzes. Moderate homework (60–90 min). Moderate expectations for productivity. Design schedule for make up work	To move to stage 4: continued symptom improvement, needs no more than one cognitive rest break per day

4	Return to full day, minimal supports (monitoring final recovery)	Attend all classes with zero to one rest breaks (20–30 min); begin modified tests (breaks, extra time). Homework (90+ min), moderate-to-maximum expectations for productivity	To move to stage 5: no active symptoms, no symptoms with cognitive or physical exertion during the full school day

5	Full return, no supports needed	Full class schedule, no rest breaks. Maximum expectations for productivity. Begin to address makeup work at this stage	N/A

N/A: Not applicable.

Adapted with permission from [4] © GA Gioia (2014).

### General accommodations

When the student is ready to return to school, several general academic accommodations can be considered to help minimize the cognitive workload (Box 1) [2,6–7,37]. The objective is to create a tolerable environment, while symptoms improve and activities are gradually resumed. Some students will quickly tolerate the full school day with minimal supports. Others may need to attend partial school days with minimal workload initially. Attending school in the morning and leaving earlier in the day may be appropriate for the student who has worsening of symptoms in the afternoon and evening, whereas the student who has difficulty falling asleep at night may benefit more by starting school mid-day, temporarily allowing him or her to sleep later in the mornings [2]. Examinations and homework assignments should be excused or delayed. A note from the medical provider may be necessary to delay certain standardized tests. Rest breaks during classes or between classes may be helpful in controlling the re-emergence of symptoms. These general academic accommodations are gradually removed as the student is able to tolerate more activity without worsening symptoms. The numbers and types of accommodations needed and their durations will vary by student.

### Symptom-specific accommodations

Certain postconcussion symptoms can be exacerbated by the school environment. Specific accommodations can help to diminish the effects of these symptom triggers (Box 2) [2,6–7,37]. The healthcare provider should request symptom-specific accommodations along with the general accommodations in the letter provided to the family and school. Concussions cause a constellation of symptoms, and numerous types of school interventions may be needed. Some of the more common symptom-specific accommodations are described here. For example, when symptoms are exacerbated by physical activities, the student can be allowed extra time to walk between classes, given an elevator pass and excused from gym class. Problems with concentration may be helped by providing the student with class notes and a seat closer to the teacher. Examinations can be taken in a quiet room, and extra time can be allowed for examinations and assignments. To alleviate noise sensitivity, the student can eat lunch in a quiet room, wear earplugs when not in class and avoid certain classes with excessive noise such as band and choir. Students who are light sensitive may benefit by wearing a hat or sunglasses, sitting in a darker part of the classroom, or turning down the brightness of computer screens. Finally, complaints of blurry or double vision can be supported by providing the student with a reading assistant or a note-taker and by minimizing computer usage.

### Chronic postconcussion symptoms

Although the majority of patients with concussion recover fully within days to weeks, a subset of patients experience prolonged symptoms that can persist for months or even years [13,46]. Using a variety of study designs, the numbers of youth with concussion who will continue to have symptoms 3 months after their injury ranges from 2.8–43% [46–53]. School management during this chronic period can be challenging for all involved. Theoretically, the management of chronic postconcussion symptoms differs from management of acute concussion. Cognitive rest is no longer the goal; school accommodations aim to promote learning during the period of chronic symptoms. The cause of persistent postconcussion symptoms is not known. Symptoms can be related to noninjury factors including premorbid psychological difficulties, family or social stressors or the presence of anxiety or depression [54–56]. When postconcussion symptoms persist for several weeks with minimal improvements, referral to a multidisciplinary concussion team is recommended. The concussion team can help to distinguish between injury-related and psychosocial factors and can work closely with the school to develop a cohesive management plan. They can also help to diagnose and treat associated symptoms that may interfere with school attendance such as headache and depression.

In most cases, school attendance should be strongly encouraged during the chronic postconcussion phase. Most students should miss only a few days of school, up to a week at most, following a concussion [4,17,57]. Prolonged school absence and decreased academic performance can be associated with reduced self-worth as well as increasing anxiety about making up missed assignments and loss of academic standing [17]. In chronic pain populations, anxiety regarding symptoms and school-related catastrophizing (e.g., thoughts such as ‘I will never be able to concentrate’ and ‘I am so far behind, I will never catch up’) have been shown to promote school avoidance [58]. In certain cases, school avoidance and prolonged school absences may perpetuate a cycle of increasing anxiety which, in turn, maintains or amplifies symptoms [58]. Breaking this cycle is crucial and often requires a multidisciplinary approach.

During the chronic recovery phase, additional academic supports may be needed. Such supports include a Section 504 Plan or an Individualized Education Plan (IEP). A ‘504 Plan’ refers to Section 504 of the Rehabilitation Act of 1973, which protects the rights of individuals who have a physical or mental impairment that limits major life activities [59]. A 504 Plan is a legally binding document that outlines reasonable accommodations that can be implemented within a general curriculum setting. A 504 Plan would be appropriate for the student who does not need modifications to the regular curriculum, such as altering the presentation of course material or changing academic testing requirements, but would benefit from accommodations such as preferential seating or taking tests in a quiet environment.

An IEP is preferred in cases where a student requires more extensive school assistance involving services above and beyond those included in the general education curriculum. The IEP is part of the Individuals with Disability Education Act (IDEA) of 2004, which is a federal law that ensures ‘free and appropriate education’ to students with a disability that ‘adversely affects the child’s educational performance and/or ability to benefit from general education’ [60]. Implementation of an IEP first requires a Multi-Factored Evaluation (MFE), which is an assessment that determines whether a student has a disability and if the disability affects educational progress. The comprehensive MFE utilizes a variety of assessment tools, including formal cognitive testing. If a child with concussion is being considered for an IEP, the medical provider should consider referral to a neuropsychologist. Formal neuropsychological testing should be considered to assess cognitive domains (i.e., attention, memory and executive functions) that are not always evaluated by school psychologists, to assess effort/symptom validity, and to assess other noninjury-related factors that can contribute to protracted postconcussion symptoms including psychosocial problems and preinjury difficulties with learning or attention [61].

### Difficulties with implementation

Several factors may hinder the successful implementation of academic accommodations. Physician letters are not legally binding documents, and schools are not obliged to offer the accommodations they recommend. School principals, athletic trainers and nurses display a range in familiarity of academic accommodations postconcussion [37,39,62]. Schools vary considerably in their staffing resources, and a variety of school personnel may be assigned to monitor concussed students and communicate with health professionals [37]. When the student has chronic postconcussion symptoms, there are reasons why implementing a 504 Plan or an IEP is preferred to using the informal recommendations from a physician letter: The 504 Plan and the IEP are legally binding and, as such, offer the student some protections; the plans help to foster communication among school personnel, families and medical providers through the formal meetings required to implement them. Given the required time and resources, the 504 Plan and the IEP each can be challenging to implement and may delay the interventions by weeks or months [59,60].

## Conclusion

Participation in school is vital to a child’s academic and social development. Effectively guiding the student with concussion through the return to school process is an important and often challenging task for the family, the medical team and the school team. School accommodations need to occur quickly, which makes it difficult to rely on traditional mechanisms for specialized education plans. Following concussion, healthcare providers and school personnel must work together to develop a return to school plan for the student that is flexible and that implements tailored academic accommodations. At this time, guidelines for school-based concussion management largely derive from expert opinion with limited empirical support. Further research is needed to determine when to return to school and how to accommodate the student during the transition. As the research efforts gain momentum, concussion education for key school and medical personnel is crucial.

## Future perspective

In the past decade, youth advocates and lawmakers successfully enacted legislation across the USA that promotes safety in youth sports and concussion education among sports participants. Over the next decade, greater focus will be placed on the student with concussion returning to school. Many questions need answers. When is the optimal time to return the symptomatic student to school? Which academic accommodations best support the transition? Which tools best promote concussion education among medical and school team participants? Future research trials that evaluate how academic interventions affect concussion outcomes will help to guide the return school process.

**Box 1.**  **General academic accommodations.**

- Allow partial school days
- Allow rest breaks during class and between classes
- Excuse missed examinations
- Excuse missed assignments
- Postpone examinations and standardized tests that cannot be excused
- Extend deadlines for assignments that cannot be excused
- Develop a feasible plan to complete missed work
- Excuse from gym class and sports until cleared by a healthcare provider
- Excuse from school activities (assemblies, field trips and after-school events) that might disrupt the daily routine or exacerbate specific symptoms

**Box 2.**  **Symptom-specific academic accommodations.**

**Poor concentration**

- Allow extra time for assignments and examinations
- Shorten assignments and reading passages
- Closely monitor classwork to verify comprehension
- Seat near the front of the room
- Provide class notes
- Allow student to take examinations in a separate, quiet room

**Fatigue, dizziness or balance problems**

- Allow an elevator pass to avoid climbing multiple flights of stairs
- Dismiss early from each class to allow extra travel time between classes
- Limit weight of book bags

**Noise sensitivity**

- Allow that lunch be eaten in a quiet room
- Excuse from band, choir or other loud classes or assemblies
- Allow ear plugs to be worn (outside of classroom)

**Light sensitivity**

- Allow hat or sunglasses to be worn
- Seat in a darker part of the classroom, away from direct sunlight
- Dim the computer screen

**Visual symptoms**

- Avoid or limit computer screen times
- Minimize reading and allow breaks while reading
- Provide a reading assistant
- Provide a note-taker or scribe

**Executive summary**

- Return to school is an essential part of youth concussion management.
- A graded return to school approach, analogous to return to play protocols, can be used.
- Academic accommodations are often warranted.
- Both general and symptom-specific accommodations can be helpful.
- Concussion education among healthcare providers and school personnel is essential to optimize the return to school process.
- Each member of the medical team and the school team must understand his or her role in supporting the returning student.
- When postconcussion symptoms persist, referral to a multidisciplinary concussion team can be helpful.
- A 504 plan or an individualized education plan may be necessary for the student with chronic postconcussion symptoms that interfere with school and learning.
