# Supplementary material for: Returning the student to school after concussion: what do clinicians need to know?
Source: Concussion. 2015 Aug 6;1(1):CNC4. doi: 10.2217/cnc.15.4 (PMC6114019; doi:10.2217/cnc.15.4)
Supplement: Supplementary file 1 [file cnc-01-4-s1.docx]

**Supplementary Material**

**Example School Letter for Academic Accommodations**

To Whom It May Concern at (School name) :

(Student) was evaluated at Nationwide Children's Hospital because he/she sustained a concussion on (injury date) . Concussions can be associated with a wide range of symptoms. The symptoms typically resolve within a days to a weeks of the injury. At this time the patient has the following postconcussion symptoms which may impact his/her learning:

( ) Cognitive difficulties (e.g. attention, concentration, memory problems, slowed thinking)

( ) Emotional difficulties (e.g. irritability, increased emotionality, anxiety, sadness, personality changes)

( ) Physical symptoms (e.g. headache, fatigue, dizziness, nausea, sleep problems, light/sound sensitivity, balance problems, vision problems)

( ) Other symptoms ________________________

( ) No symptoms

**Attendance.** We recommend:

( ) Full school days as tolerated

( ) Partial school days, may advance to full school days as tolerated

( ) Partial school days, until cleared to advance by a medical provider (clinic follow up date _______)

( ) No school until cleared by a medical provider (clinic follow up date _______)

**Physical Activity Restrictions** until further cleared by a healthcare provider

( ) No physical activity during recess or school

( ) No PE class

( ) No participation in contact sports

**Recommended Accommodations:** The following accommodations are suggested for school. These are expected to be short-term in nature and can be lifted as tolerated, unless otherwise stated.

( ) Extended time on tests and assignments

( ) Reduced workload (reduce amount of make-up work or homework; shorten assignments)

( ) Preferential seating in classroom

( ) Testing in quiet environment

( ) Postpone tests if possible

( ) Copies of notes or outlines

( ) Allow breaks as needed

( ) Reduce sensory stimulation (allow sunglasses and/or ear plugs, reduce time in loud environments)

( ) Limit time on computer screens

( ) Other _________________________________________________________________________

________________________________________________________________________________

________________________________________________________________________________

(Provider’s name, contact information, signature, and today’s date)
